# Systematic Review of the Literature About the Effects of the COVID-19 Pandemic on the Lives of School Children

**DOI:** 10.3389/fpsyg.2020.569348

**Published:** 2020-10-14

**Authors:** Javier Cachón-Zagalaz, María Sánchez-Zafra, Déborah Sanabrias-Moreno, Gabriel González-Valero, Amador J. Lara-Sánchez, María Luisa Zagalaz-Sánchez

**Affiliations:** Department of Didactics of Musical, Plastic and Corporal Expression, University of Jaén, Jaén, Spain

**Keywords:** children, COVID-19, coronavirus, physical activity, psychology

## Abstract

**Background:** The year 2020 has been marked by the emergence of coronavirus disease 2019 (COVID-19). This virus has reached many countries and has paralyzed the lives of many people who have been forced to stay at home in confinement. There have been many studies that have sought to analyze the impact of this pandemic from different perspectives; however, this study will pay attention to how it has affected and how it may affect children between 0 and 12 years in the future after the closure of schools for months.

**Objective:** The objective of this article is to learn about the research carried out on the child population in times of confinement, especially those dealing with the psychological and motor aspects of minors.

**Methods:** To carry out this systematic review, the PRISMA statement has been followed to achieve an adequate and organized structure of the manuscript. The bibliography has been searched in the Web of Science (WOS), Scopus, and Dialnet databases, using as keywords: “COVID-19” and “Children.” The criteria that were established for the selection of the articles were (1) articles focusing on an age of up to 12 years, (2) papers relating COVID-19 to children, and (3) studies analyzing the psychological and motor characteristics of children during confinement.

**Results:** A total of nine manuscripts related to the psychological and motor factors in children under 12 have been found. The table presenting the results includes the authors, title, place of publication, and key ideas of the selected manuscripts.

**Conclusion:** After concluding the systematic review, it has been detected that there are few studies that have focused their attention on the psychological, motor, or academic problems that can occur to minors after a situation of these characteristics. Similarly, a small number of studies have been found that promote actions at the family and school level to reverse this situation when life returns to normal. These results may be useful for future studies that seek to expand the information according to the evolution of the pandemic.

## Introduction

When news of an epidemic began to spread in a Chinese city in early 2020, no one anticipated the scope of the epidemic for the entire world in a very short period. From Wuhan (China) to New York (USA) through Africa, South America, Asia, and Europe, the new coronavirus, coronavirus disease 2019 (COVID-19) or severe acute respiratory syndrome coronavirus 2 (SARS-CoV-2), has paralyzed, to a greater or lesser extent, the life in many countries, causing thousands of deaths and about 6 million infections. For these reasons, the scientific community is on the alert by conducting studies on the virus, the disease it produces, the situation it creates, and the population it attacks, from different perspectives, including systematic reviews of the literature, such as the one presented in this paper.

However, researchers on this topic are not only biologists or physicians. It is worth noting the contribution of Maestre Maestre (2020), President of the Society for Latin Studies, in an article on the virus that has caused the pandemic, in which, playing with different related terms, he explains that the neutral noun “virus” means “poison” in Latin, so most current research is trying to find a medicine that will kill the virus. Likewise, the Greek term ϕάρμακoν (in Latin pharmacum) also means poison. The relationship between the two terms is that pharmacies are looking for poisons that will kill the “poisons” that undermine people's health or their desire to be safe. Remember the symbol of the pharmacies, the “Bowl of Hygieia” with the snake that pours a “poison” into it that stops being a poison to become an antidote. The name “coronavirus” is given to it because, through the microscope, the “virus-poison” is shaped like a “crown” that makes it king of poisons.

However, in addition to scientists who study the pandemic, biologists, doctors, and humanists, educators are obliged to care for the psychological and emotional health, as well as cultivate the minds, of children. The consequences of the containment measures of COVID-19 are being detrimental to the mental health of people around the world. It is logical that the most vulnerable are children who do not understand what is happening and who, along with the concern and frustration of their elders, may present risk factors, such as anxiety and affective and post-traumatic stress disorders (Giallonardo et al., 2020). However, not only minors are affected. According to Roy et al. (2020), more than 80% of people over 18 have shown the need for attention to their mental health as a result of the anxiety and stress experienced during the pandemic. Forte et al. (2020) agree with this idea, stating that the pandemic has caused stress, psychological discomfort, sleep disorders, and instability, among others, in a large part of the population.

In this sense, many questionnaires have been applied to obtain information in the educational context or related to it from research groups at different universities, including the one from the IDIBAPS research group at the Hospital Universitario de Barcelona, concerning behaviors to reduce emotional distress during the pandemic and confinement by COVID-19, https://enquesta.clinic.cat/index.php/268395?lang=es; Universidad de las Palmas de Gran Canaria on family relationships during confinement: Study of the effect of COVID-19 in the family context, https://forms.gle/2xpmqRtQ8mtBMAz77; Universidad de Oviedo, as a longitudinal study on how isolation and the practice of physical activity (PA) during confinement is affecting to offer effective strategies that it called “pills”: EDAFIDES Questionnaire COVID-19, https://docs.google.com/forms/d/e/1FAIpQLSfyID6X7YgUejwXNv2YyOQ1YU2LrFsPkkvHzux_TD_BjPIGNw/viewform?usp=sf_link; Euskal Herriko Unibertsitatea, to find out about the situation of university students in confinement and to propose improvements: https://forms.gle/jDkFgW7xeKfSFNHB6; Universidad da Coruña y Universidad de Jaén, on the activities of children in Spanish homes in times of confinement. This last questionnaire was applied in Spain and in South America: https://docs.google.com/forms/d/e/1FAIpQLSeyBBkMEmPxj-AoPQG98QorsaLyNex9wlI2FJ2Ku2q8nbsdNQ/viewform.

Based on the above-mentioned questionnaires, there is a concern to analyze how confinement has affected children under 12 at the motor and psychological levels. This literature review is carried out and explained in detail in the procedure and search strategy of the methodology. The impact of the pandemic is such that many national and international journals are offering special issues on COVID-19, including Frontiers, which, being digital, contains 229 articles signed by many authors from various countries, which look at the subject from different perspectives: there are eight that refer to age and especially to children in some way, including: who cares about the elderly (Fischer et al., 2020), physical inactivity (Ricci et al., 2020), age distribution (Cortis, 2020), and newborns (Ovali, 2020), but none discusses parents' views on the period of confinement from the psychological, educational, academic, physical, and emotional points of view of their children. Neither do they inquire into the opinion of the children themselves, understanding by these those who are in infant and primary education, that is, up to the age of 12.

Education must seek to provide the child with a comprehensive education, trying to help his or her physical, emotional, intellectual, family, social, and moral development. Active methods are crucial for early childhood education, and teachers are needed to apply them in schools (Salvador, 2008), now in the homes of their students, which they access through the Internet. The role of parents is also to educate, but from different perspectives, complementing those of teachers in the acquisition of children's learning. For these reasons, many families say that they do not know how to undertake these activities with their children for so long.

Likewise, the lack of other family members, such as grandparents, who had been playing a role in accompanying, especially with children in preschool, complicates the state of confinement and the lack of school attendance that is taking place, initially planned for 6 months in a row. The study by Clemente-González (2016) of the University of Murcia highlights the relevance of grandparent–grandchild relationships and the role of the former in the social and emotional development of the child, which gives great significance to their grandparents for the appreciation observed in them, recognizing their importance in the family structure. At this point, it is also necessary to point out the lack of relationships between equals, which is so important for the correct emotional development of children.

Another important aspect that has been affected by the coronavirus pandemic is the practice of PA. Many schoolchildren practice physical exercise based solely on the subject of Physical Education. This subject is not only based on motor skills but is a practice that affects schoolchildren in a global way, influences many aspects of their daily lives, and helps teachers to better understand students in their different dimensions (Founaud and González-Audicana, 2020). Lack of PA is associated with obesity, as indicated by different studies that relate the regular practice of physical exercise with the reduction of health problems (Castañeda-Vázquez et al., 2020).

The opinion article written by the Spanish secondary school teacher, Fandino-Pérez (2020), is significant in which he reflects on the virtuality of education and his position regarding personalized education, so demanded in times of normality, where teachers and students know each other, interact, and socialize, precisely the attitude that has taken away the virus. Fandino-Pérez says that the pandemic has put us in front of the mirror to see a distorted and absurd image of the work of teachers as producers of programming and good results, which turns them and their students into a kind of machine. We have forgotten the main thing: to be human beings capable of creating a better world and of overcoming ignorance, fear, and demagogy.

As a background to this study, we refer to March 11, 2020 when the World Health Organization (World Health Organization, 2020a) declared this disease produced by the coronavirus (COVID-19) to be a pandemic. It was first reported in Wuhan (China) on December 31, 2019. According to World Health Organization (2009), the global public health community recognized the need for standardized research and data collection after the 2009 flu epidemics, so the WHO Expert Working Group on Special Research and Studies has developed several standard protocols for pandemic flu. This has led World Health Organization (2019a, 2020b) to develop similar protocols for the Middle East respiratory syndrome coronavirus (MERS-CoV) and, with the support of expert advisors, has adapted the protocols for influenza and MERS-CoV to help better understand the clinical, epidemiological, and virological characteristics of COVID-19.

Some months have passed, and most of the inhabitants of planet Earth, more or less surprised, have been confined to their homes for about 60 days, where they have carried out their work online and have had to attend to their younger children, also confined without attending school and without being able to go out into the street or use the recreational facilities that some residential areas have.

When we find ourselves at the moment of reincorporation into the daily life known before the appearance of the pandemic (May 2020), other illnesses arise as a consequence of the involuntary confinement to which the population has been subjected; this is the cave syndrome or agoraphobia (fear of open spaces), and it is possible that with the passage of time, other psychological and affective disorders will arise in the adults who will be those who have suffered this confinement and this disaster as children.

The disease mainly attacks people over 70 years old and only 0.3% of children in countries where there have been more deaths (for example, Spain). According to the Instituto de Salud Carlos, this may be the reason why medical research does not deal with children, but these subjects have special psychological, academic, and emotional characteristics at a stage of their lives when they are in full development, so from the educational point of view, it is necessary to find out how children have developed in their homes, what their parents think, and what future expectations experts, teachers, and psychologists have for them.

For all these reasons, the aim of this work is to find out about the research carried out on the child population in times of confinement, especially those that deal with the psychological and motor aspects of minors.

Considering this objective and following the Population, Intervention, Comparison, and Outcome (PICO) strategy, the following research question arises: what do the studies already published determine about how confinement has affected children under the age of 12 on a psychological and motor level?

## Methodology

For the elaboration of this systematic review, we have followed the items to publish systematic reviews and meta-analyses of the PRISMA statement (Sotos-Prieto et al., 2014; Hutton et al., 2015), in order to achieve an adequate and organized structure of the manuscript. The guidelines of Cochrane Training (Higgins and Green, 2011) have also been used.

### Procedure and Search Strategy

The literature review took place during the last weeks of May 2020 and focused mainly on the Web of Science (WOS) database, using Scopus and Dialnet as support. The topic considered for the selection of articles was the one related to the global pandemic caused by COVID-19 and how it has affected psychologically and motorically children up to 12 years old. The following keywords were used: “COVID-19” and “children” and the Boolean operator “and.” After this first search and taking into account only the works published in 2020 (since that is when the pandemic occurred), 837 scientific documents were obtained. By restricting the search to only journal articles, the documents were reduced to 576 articles, after which the language filter was applied, selecting only those papers published in English and Spanish, leaving a total of 537. Since the pandemic started in China, the initial search was also done in that language, not finding any related articles. The articles signed by researchers of Chinese nationality are written in English. Finally, the following areas of research were chosen: “Psychology,” “Sociology,” and “Education Educational Research,” finally limiting the search to 48 scientific articles, which make up the sample of this study.

### Inclusion and Exclusion Criteria

The criteria that were established for the selection of the articles were (1) articles focusing on an age of up to 12 years, (2) papers relating COVID-19 to children, and (3) studies analyzing the psychological and motor characteristics of children during confinement.

**Figure 1:**
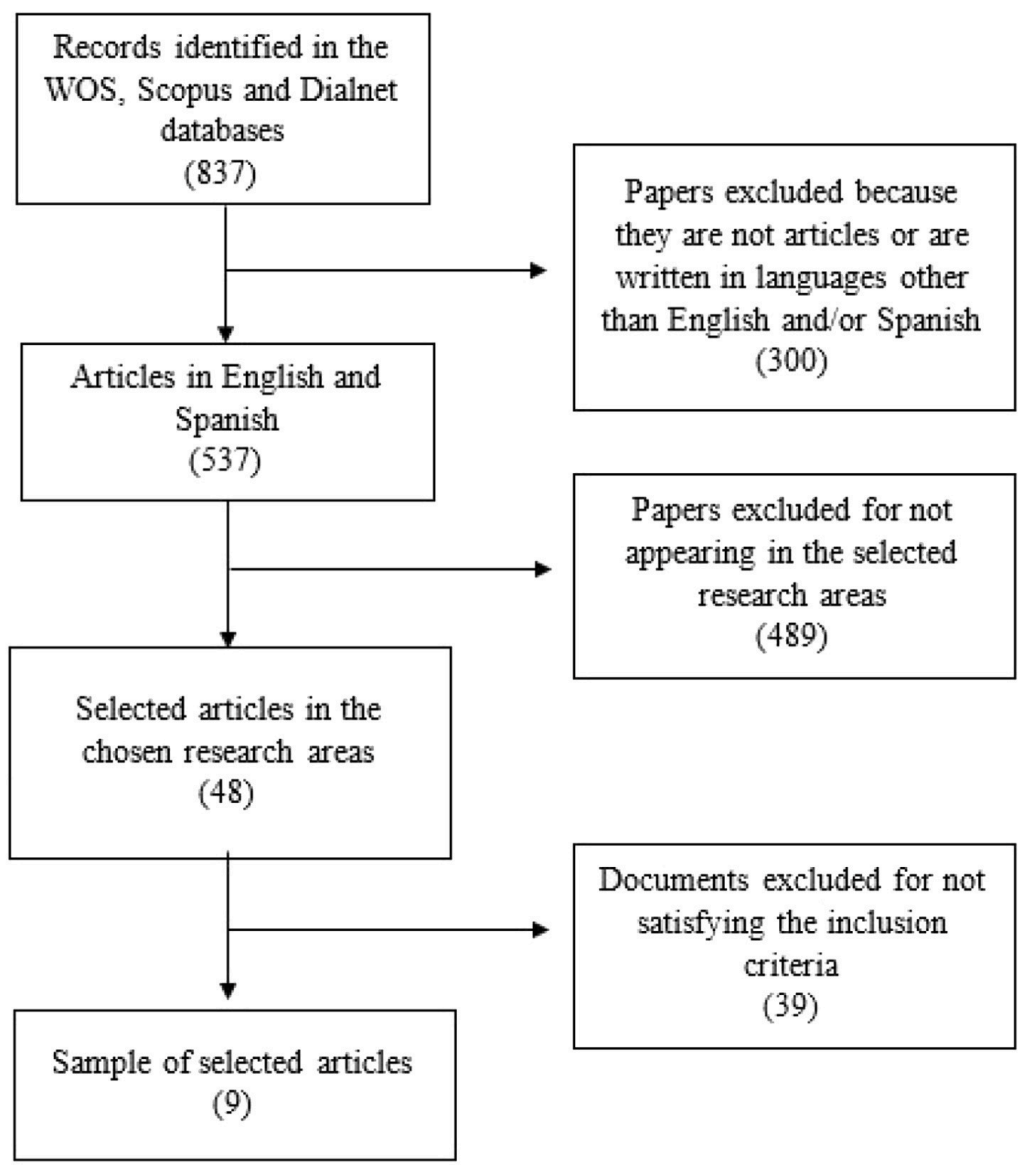
PRISMA flowchart.

In order to apply these criteria, a first preliminary reading of the title and summary of each article was carried out, which made it possible to rule out papers that did not meet the above-mentioned criteria. A more exhaustive reading of the selected articles was then carried out, leaving a final sample of nine scientific papers (Figure 1).

### Article Coding

To extract the data from the articles, the following coding process was followed: (1) author/authors and year of publication, (2) title of the research, (3) place/country of publication, and (4) key ideas of the research.

The research included in this systematic review was coded by four of the authors, in order to check the reliability of the coding and the degree of agreement among the researchers in relation to the selection and extraction of the data (González-Valero et al., 2019). The degree of agreement on the rating of the articles was 93%. This was obtained by dividing the number of coincidences by the total number of categories defined for each study and multiplying it by 100.

In order to establish the methodological quality of the present study, reliability was determined according to the detection and selection of the Fleiss' Kappa (Fk) statistical index for more than two evaluators (Fleiss, 1971). A value of Fk = 0.780 was obtained for data extraction and selection, which indicates that there is substantial agreement (0.61–0.80).

Table 1 presents the main results of different studies following the codification indicated in the previous section: (1) author/authors and year of publication, (2) title of the research, (3) place/country of publication, and (4) key ideas of the research.

**Table 1:** Basis of the study.

**Authors and year**	**Title**	**Country**	**Key ideas**
Szabo et al. (2020)	From helpless to hero: promoting values-based behavior and positive family interaction in the midst of COVID-19	USA	- Importance of the role of parents in the confinement of their children. - It is necessary for parents to establish schedules and routines to achieve psychological stability for their children. - Tips are proposed to make confinement easier for children
Dalton et al. (2020)	Protecting the psychological health of children through effective communication about COVID-19	UK	- Psychological consequences that confinement can have on children. - Children are exposed to large amounts of information and may not know how to handle it. Parents have to explain the situation to them, taking into account their age, making them see that they are not to blame for the situation. - Children may show distress, guilt, feel threatened, worry…. - They miss their other caregivers (e.g., grandparents)
Yarimkaya and Esentürk (2020)	Promoting physical activity for children with autism spectrum disorders during coronavirus outbreak: benefits, strategies, and examples	Turkey	- It focuses on children with autism spectrum disorder (ASD). - It deals with the importance of PA during confinement. - It proposes exercises that these children with ASD can do during the time they are locked up in the house
Liu et al. (2020)	Mental health considerations for children quarantined because of COVID-19	China	- It focuses on children who are separated from their families or caregivers because one or the other is infected with coronavirus. - These children are at risk for acute stress, adjustment disorder, and grief. - Children who are isolated because they are infected with the coronavirus may suffer from post-traumatic stress. - Children who have lost their parents to this infection may commit suicide as adults as a result. - As for “normal” home confinement with parents, they mention that it can cause stress in children, although being with their parents can relieve it
Ricci et al. (2020)	Recommendations for physical inactivity and sedentary behavior during the coronavirus disease (COVID-19) pandemic	Italy	- It focuses on the inactivity and sedentariness that the coronavirus has brought to the world population and its consequences on the health of individuals. - It presents PA recommendations for the entire population, also specifically mentioning exercise for children
Guan et al. (2020)	Promoting healthy movement behaviors among children during the COVID-19 pandemic	China	- Reminder of the worldwide recommendations on daily PA time in children. - Child sedentarism as an effect of confinement. - Increased use of digital technologies. - Recommendations to parents and caregivers for the promotion of daily healthy behaviors
Zhang et al. (2020)	Acute stress, behavioral symptoms and mood states among school-age children with attention-deficit/hyperactive disorder during the COVID-19 outbreak	China	- Worsening behavior in children with attention-deficit/hyperactive disorder during confinement. - Stress levels experienced by family members and children with this disorder
Álvarez-Zarzuelo (2020)	El confinamiento de niñas y niños En España en 2020 por la Crisis del COVID-19: Propuestas desde la Educación Social Escolar para la vuelta al centro escolar	Spain	- Personal opinion article. Social educator concerned with how confinement will affect children psychologically. - Digitally illiterate or financially unsound families will create an academic gap among children. - Compilation of 12 needs of confined minors and responses at the socio-educational level to address them on their return to the classroom
Gómez-Gerdel (2020)	El cerebro pleno del niño/a: la labor de un/a maestro/a de educación inclusiva con las familias en tiempos de confinamiento. Una reflexión educativa	Spain	- Crisis in the Spanish educational system originated by the COVID-19 pandemic, consequence: virtual education. - Benefits of confinement: possibility for minors to acquire greater autonomy in daily household tasks and improvement in family relations by living together with parents and children for longer periods of time. - Inclusive education in confinement and its difficulty in alleviating inequalities. - Self-knowledge and understanding of emotions and actions. - Promotion of correct coexistence with children in confinement and techniques for the integration of the upper and lower brain

## Discussion

Of the nine articles analyzed because they met the characteristics of the search, three have been published in *The Lancet*, which began as an independent international weekly medical journal, founded in 1823 by Thomas Wakley. Since its first issue, it has strived to make science widely available so that medicine can serve, transform society, and positively impact people's lives. It has evolved into a family of journals including *The Lancet Child & Adolescent Health*, in which one of the three articles cited appears. These three articles, and most of those analyzed, relate to the classical medicine that should serve society to help improve life.

Most of the references in this article (84.22%) are from the year 2020, a sign of the interest in the subject and the dedication of scientists and teachers. Only three are earlier, the one by Hutton et al. (2015) that deals with a more technical content, the extension of PRISMA for network meta-analysis, and the ones by Salvador (2008) and Clemente-González (2016) that highlight the role of grandparents in children's lives.

Of the two articles by Spanish teachers, the one by Álvarez-Zarzuelo (2020) is a personal opinion of a social educator who is ahead of other research. It only provides the experts' ideas on the possible repercussions of confinement. For his part, Gómez-Gerdel (2020) writes an opinion article that, exceptionally, is being published by the International Journal of Education for Social Justice in its special issue 9(e) on “Consequences of the Closure of Schools by COVID-19 on Educational Inequalities.” The author, from the perspective of the departments of Educational Guidance that deal with inclusive education, raises the chaos that it has meant for the Spanish Educational System to apply teaching only on line, which means for the most vulnerable families: difficulties in accessing technologies and delays in education. On the other hand, it raises what could be a return to the family whose members had been living together for a long time, something absolutely necessary for the correct development of the minors who spend too much time away from home.

The teaching–learning system, which should seek the comprehensive training of the child, in which parents and teachers should participate, has been drastically modified, trying not to abandon the active methods used in schools (Salvador, 2008), with the difficulties that this entails for families, which in many cases have no training in this area.

Of the three articles by Chinese authors, Liu et al. (2020) analyze the situation of children whose parents have been infected with the virus or have died; Zhang et al. (2020) observe the behavior of children with attention-deficit/hyperactive disorder (ADHD) during this period; and finally, Guan et al. (2020) deal with the practice of childhood PA during confinement. Therefore, only one of them studies a type of activity in this period, the one dealing with PA coinciding with what is written by the Italians Ricci et al. (2020); in the same line, we find the Turks Yarimkaya and Esentürk (2020) who deal with the importance of PA in confinement for children with autism spectrum disorder (ASD). It is important to remember that World Health Organization (2010, 2019b) recommends a minimum of 1 h/day of moderate–vigorous PA in children, but that only one-third of children exceed these recommendations (Salas-Sánchez et al., 2020).

The American and British authors analyze the role of parents in the confinement of their children and provide some advice on this subject. They also look at the future psychological problems that may arise as a result of over-information, change of routines, and manifestation of feelings of distress and guilt, as well as the need to see peers and other carers (teachers, grandparents). They coincide with Clemente-González (2016) project based on the grandparent–grandchild relationship and the promotion of identity, which seemed to be a premonition of what would happen with the arrival of the COVID-19 pandemic that would force the disappearance of these relationships for a long time.

It is important to note that, according to the review carried out, there are authors who analyze the pandemic from different perspectives with which we agree: cultural aspects (Maestre Maestre, 2020); actions of biologists and doctors, more distant from our intentions; humanists (Fandino-Pérez, 2020), and especially for this study, of educators who are aware that the essence of being in the classroom and the immediate feedback that students offer in this situation has been lost. To this must be added the role of the WHO, overwhelmed by the health events that have occurred so quickly, as described in these lines.

We believe that the application of many questionnaires during the confinement and currently post-COVID-19 pandemic has saturated the patience of the respondents, although most have helped scientists and educators to obtain information that will facilitate a smooth exit from this disaster.

## Conclusions

The above leads us to the general conclusion that there are very few studies on how confinement has affected children under 12 years old psychologically and motorly. These articles agree on the consequences that confinement can have on minors and on the importance of psychological support from the family, and the establishment of routines can be effective. The manuscripts that deal with PA remind us of the importance of it and indicate that the rates of sedentarism have increased during these months.

It is necessary to insist on the search for and analysis of other activities, as well as the behavior of parents and children in these circumstances, in order to prevent possible psychological and academic problems and because if the online teaching situation is prolonged, it is very important to know how to act from the educational and family environment.

The main limitation the authors have faced has been the small number of scientific articles related to the area of study. This scarcity of published works makes it necessary to continue researching this. This is the reason why our study can serve as a starting point or theoretical foundation for further studies.

## Author Contributions

JC-Z, MS-Z, DS-M, GG-V, AL-S, and MZ-S contributed to the conception and design of the revision. All authors wrote some part of the manuscript and all reviewed the manuscript.

## Conflict of Interest

The authors declare that the research was conducted in the absence of any commercial or financial relationships that could be construed as a potential conflict of interest.
